# Catalpol protects rat ovarian granulosa cells against oxidative stress and apoptosis through modulating the PI3K/Akt/mTOR signaling pathway

**DOI:** 10.1042/BSR20194032

**Published:** 2020-04-17

**Authors:** Jin Yan, Disi Deng, Yeke Wu, Keming Wu, Jie Qu, Fei Li

**Affiliations:** 1 Hospital of Chengdu University of Traditional Chinese Medicine, Chengdu, 610072, Sichuan Province, P.R. China; 2 Department of Stomatology, Hospital of Chengdu University of Traditional Chinese Medicine, Chengdu, 610072, Sichuan Province, P.R. China; 3 Department of Gynaecology, Hospital of Chengdu University of Traditional Chinese Medicine, Chengdu, 610072, Sichuan Province, P.R. China; 4 Shaanxi University of Chinese Medicine, Xianyang 712046, Shaanxi Province, P.R. China

**Keywords:** apoptosis, catalpol, disrupted follicular development, granulosa cells (GCs), oxidative stress, PI3K/Akt/mTOR signaling pathway

## Abstract

Disrupted follicular development may result in increased follicular atresia, which is a crucial mechanism of various ovarian pathologies. It has been demonstrated that oxidative stress is associated with disrupted follicular development. Catalpol is a natural compound that has been found to possess antioxidative stress. However, the effects of catalpol on oxidative stress-induced disrupted follicular development remain unclear. In the present study, we evaluated the protective effect of catalpol on hydrogen peroxide (H_2_O_2_)-induced oxidative damage in granulosa cells (GCs), which play crucial roles in the follicular development. Our results showed that catalpol significantly improved cell viability, reduced reactive oxygen species (ROS) and malondialdehyde (MDA) production, and elevated superoxide dismutase (SOD) and glutathione peroxidase (GSH-Px) activities in H_2_O_2_-induced GCs. Catalpol treatment caused significant increase in bcl-2 expression, and decreases in bax and caspase-9 expressions. Compared with the H_2_O_2_-induced GCs, caspase-3 activity in catalpol-treated cells was markedly decreased. Furthermore, catalpol caused significant activation of PI3K/Akt/mTOR pathway in GCs in response to H_2_O_2_ stimulation. Additionally, inhibition of this pathway reversed the inhibitory effects of catalpol on H_2_O_2_-induced oxidative injury and apoptosis in GCs. In conclusion, these findings suggested that catalpol protected GCs from H_2_O_2_-induced oxidative injury and apoptosis via activating PI3K/Akt/mTOR signaling pathway. Thus, catalpol might serve as a therapeutic approach for regulating disrupted follicular development.

## Introduction

Follicular development is a complicated physiological progress, which is essential for a functional ovary and fertility [1]. Follicular development starts with the activation of resting follicles, and gradually leads to the growth and selection of dominant follicles (DFs) from small health follicles [2,3]. It has been demonstrated that disrupted follicular development may result in increased follicular atresia, which is a crucial mechanism of various ovarian pathologies such as endometriosis and polycystic ovary syndrome (PCOS) [4]. Therefore, understanding the molecular mechanism of follicular development is essential for the treatment of ovarian pathologies.

Granulosa cells (GCs) are a group of cells located outside the zona pellucida in an individual follicle [5]. GCs are closely associated with the complex transition from primordial follicles to mature follicles [6]. In recent years, there has been growing interest in the roles of oxidative stress in female reproduction. Accumulating evidence demonstrates that reactive oxygen species (ROS) production is markedly increased in response to diverse stimuli, such as exogenous toxicants and ionizing radiation [7]. ROS are key signals in the initiation of oxidative stress and apoptosis of GCs, leading to dysregulation of follicular development [8]. Thus, attenuating ROS-mediated oxidative stress and apoptosis in GCs may be novel approach for maintaining normal follicular development.

Catalpol is an iridoid glycoside extracted from *Rehmannia* root and has been reported to possess broad activities, especially antioxidative effect [9,10]. Catalpol protects pre-myelinating oligodendrocytes (PreOLs) against ischemia-induced oxidative injury through ERK1/2 signaling pathway [11]. Catalpol protects against hydrogen peroxide (H_2_O_2_)-induced oxidative stress in astrocytes primary cultures via reducing intracellular ROS formation and preventing the decrease in the activities of antioxidant enzymes [12]. Additionally, catalpol may be a candidate agent for the treatment of oxidative stress-induced neurodegenerative disease [13]. However, the direct protective effects of catalpol on follicular development and the underlying molecular mechanisms remain unclear. Thus, the aim of the present study was to evaluate the effect of catalpol on H_2_O_2_-induced oxidative stress and apoptosis in GCs.

## Materials and methods

### Cell culture

Wistar rats (Beijing Vital Laboratory Animal Technology, Beijing, China) used in the present study were maintained in a room with controlled illumination (lights on: 7–21 h), temperature (26–28°C)and humidity (60 ± 2%) with free access to regular rat diet and water. The animal work took place in Animal Centre of Chengdu University of Traditional Chinese Medicine. The animal experiments were approved by the Animal Care and Use Committee of Hospital of Chengdu University of Traditional Chinese Medicine (Chengdu, China).

GCs were prepared as described previously with some modifications [14]. Briefly, immature female rats (21–27 days old) were injected intraperitoneally with 10 U of pregnant mare serum gonadotropin (PMSG) to stimulate follicular development. Then the rats were anesthetized with sodium pentobarbital (40 mg/kg body weight) and the ovaries were removed. GCs were isolated using a non-enzymatic needle puncture method to release the cells from follicles. The cells were plated and cultured in DMEM (supplemented with 100 U/ml penicillin, 100 U/ml streptomycin and 2 mM l-glutaminate) containing 10% FBS and incubated at 37°C.

### Lactate dehydrogenase release assay

GCs were seeded in 96-well plates (6000 cells/well) and cultured for 24 h. Then, cells were treated with catalpol at different concentrations ranging from 5 to 40 μM for 24 h. After treatments, lactate dehydrogenase (LDH) activity in culture medium was determined using an LDH cytotoxicity assay kit (Promega, Madison, WI, U.S.A.) according to the protocol.

### Cell viability assay

The CCK-8 assay was performed to assess cell viability of GCs after treatment with 0, 5, 10 and 20 μM of catalpol. After incubation, CCK-8 solution (10 µl; Dojindo, Kumamoto, Japan) was added to each well and incubated for additional 4 h at 37°C. The OD values in each group were recorded using a microplate reader (Bio-Tek, Winooski, VT, U.S.A.) at 450 nm.

### Detection of ROS level

The level of oxidative stress was monitored by the measurement of ROS production using a fluorescent probe H_2_-DCFDA (Sigma–Aldrich, St. Louis, MO, U.S.A.), which can be rapidly oxidized to become highly fluorescent DCF in the presence of ROS. GCs with different treatments were incubated with 4 μM of H_2_-DCFDA at 37°C for 40 min. The cells were observed using an inverted fluorescence microscope and the fluorescence intensities were analyzed by Image-Pro Plus 6.0 software (Media Cybernetics, Bethesda, MD, U.S.A.).

### ELISA

The levels of antioxidant and apoptotic markers including malondialdehyde (MDA), superoxide dismutase (SOD) and glutathione peroxidase (GSH-Px) in culture supernatants of GCs were determined with commercial ELISA kits (Nanjing Jiancheng Bioengineering Institute, Nanjing, China) according to the protocol.

### Detection of caspase-3 activity

Caspase 3 activity was detected with Caspase 3 activity assay kit (Beyotime) per the manufacturer’s instructions. Briefly, GCs were lysed and incubated with the substrate Ac-DEVD-pNA (2 mM) at 37°C for 1 h. Then the absorbance values were read at 405 nm.

### Western blot analysis

GCs were harvested and resuspended in RIPA lysis buffer (containing 1 mM PMSF). After incubation for 30 min on ice, the mixtures were centrifuged at 14000×***g*** for 10 min. Protein concentration in the supernatants were measured using BCA protein assay. Each sample (containing 30 μg protein) was mixed with 5× loading buffer and boiled at 100°C for 5 min. Proteins were separated by 12% SDS/PAGE and electrophoretically transferred on to polyvinylidene difluoride (PVDF) membranes (Bio-Rad Laboratories, Hercules, CA, U.S.A.). The membranes were blocked with 5% (w/v) non-fat milk in TBST buffer for 2 h to block non-specific protein-binding sites. Subsequently, the membranes were incubated overnight with rabbit monoclonal antibody against bax, bcl-2, caspase-9, PI3K, p-PI3K, Akt, p-Akt, mTOR, p-mTOR or β-actin (Abcam, Cambridge, MA, U.S.A.). After that, the membranes were incubated with horseradish peroxidase (HRP)–conjugated goat anti-rabbit IgG (1:1000; Abcam) in TBST for 1 h at room temperature. After washing for three times, the protein bands were visualized using ECL Western blotting substrate kit (Thermo Fisher Scientific, Waltham, MA, U.S.A.) and quantitated applying a Quantity One image densitometer (Bio-Rad).

### Statistical analysis

All experimental data were expressed as means ± standard deviation (SD) from at least three repeated experiments. Statistical analysis was performed using the SPSS version 13.0 software (SPSS Inc., Chicago, IL, U.S.A.). Statistical significance among multiple groups was determined by one-way Analysis of Variance (ANOVA). Differences were considered significant for *P*<0.05.

## Results

### Cytotoxicity effect of catalpol on GCs

**Figure 1:**
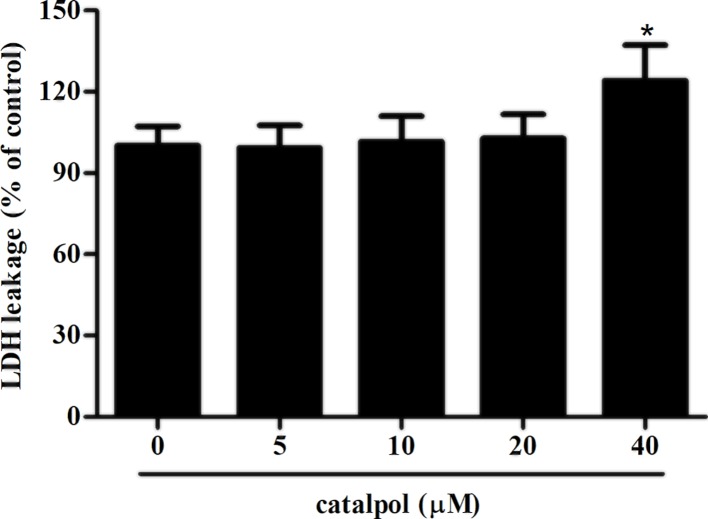
LDH release assay was used to determine the cytotoxicity effect of catalpol on GCs GCs were treated with catalpol at different concentrations ranging from 5 to 40 μM for 24 h. LDH activity in culture medium was determined using an LDH cytotoxicity assay kit. *P<0.05 versus the control GCs.

To detect the cytotoxicity effect of catalpol on GCs, GCs were treated with catalpol at different concentrations ranging from 5 to 40 μM for 24 h. LDH cytotoxicity assay showed that catalpol did not exhibit cytotoxicity effect on GCs even in the concentration of 20 μM (Figure 1). Therefore, we selected the concentrations of 5, 10 and 20 μM for the following experiments.

### Catalpol improved cell viability in H_2_O_2_-stimulated GCs

**Figure 2:**
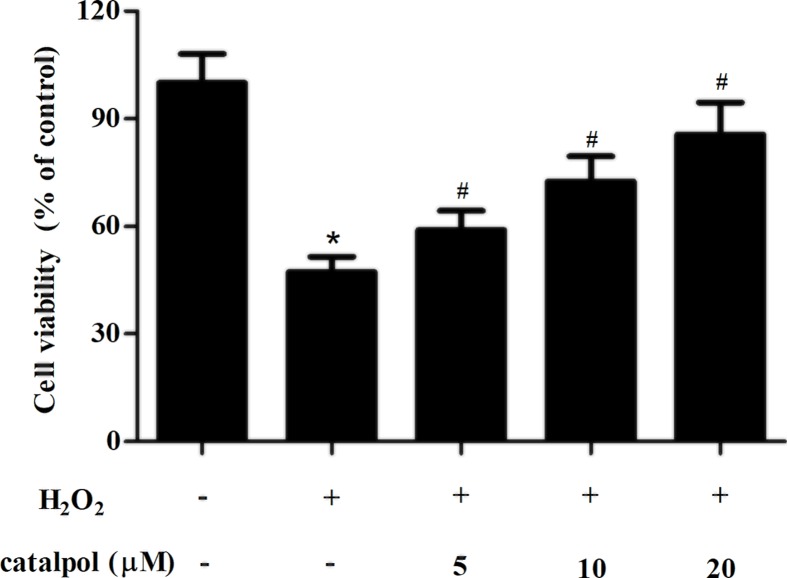
Effect of catalpol on cell viability in H_2_O_2_-stimulated GCs GCs were pretreated with 5, 10 and 20 μM of catalpol for 2 h, and then stimulated with H_2_O_2_ (150 μM) for 24 h. Cell viability was measured using CCK-8. *P<0.05 versus the control GCs. ^#^*P*<0.05 versus the H_2_O_2_-stimulated GCs.

Then, we used H_2_O_2_ to stimulate oxidative injury in GCs. GCs were pretreated with 5, 10 and 20 μM of catalpol for 2 h, and then stimulated with H_2_O_2_ for 24 h. As shown in Figure 2, compared with the control group, cell viability in H_2_O_2_ stimulation group was markedly reduced. However, treatment with catalpol (5, 10 and 20 μM) resulted in dose-dependent increase in cell viability.

### Catalpol inhibited oxidative stress in H_2_O_2_-stimulated GCs

**Figure 3:**
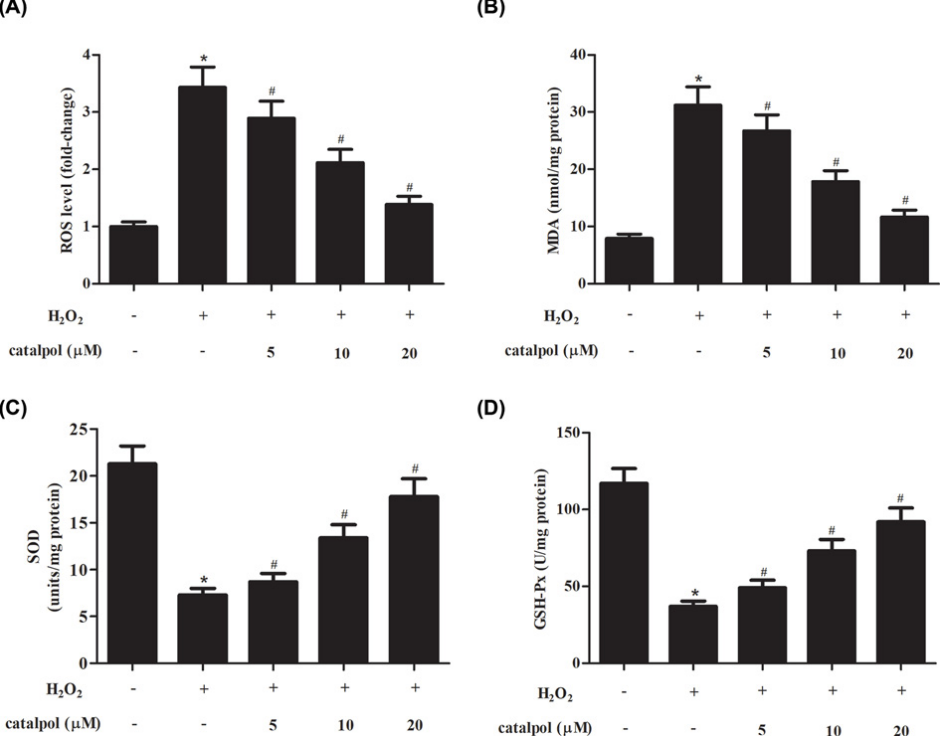
Effect of catalpol on oxidative stress in H_2_O_2_-stimulated GCs GCs were pretreated with 5, 10 and 20 μM of catalpol for 2 h, and then stimulated with H_2_O_2_ (150 μM) for 24 h. (**A**) The ROS production was determined using a fluorescent probe. (**B**–**D**) The levels of MDA, SOD and GSH-Px were determined using ELISA. *P<0.05 versus the control GCs. ^#^*P*<0.05 versus the H_2_O_2_-stimulated GCs.

The production of ROS and MDA, and the activities of SOD and GSH-Px were evaluated as markers of oxidative stress. As shown in Figure 3A, the intracellular ROS production was markedly increased in GCs exposed to H_2_O_2_ stimulation. While the increased ROS production was attenuated by catalpol in a dose-dependent manner. ELISA proved that H_2_O_2_-caused increase in MDA level and decrease in SOD and GSH-Px activities were mitigated by treatment with catalpol (Figure 3B–D).

### Catalpol inhibited cell apoptosis in H_2_O_2_-stimulated GCs

**Figure 4:**
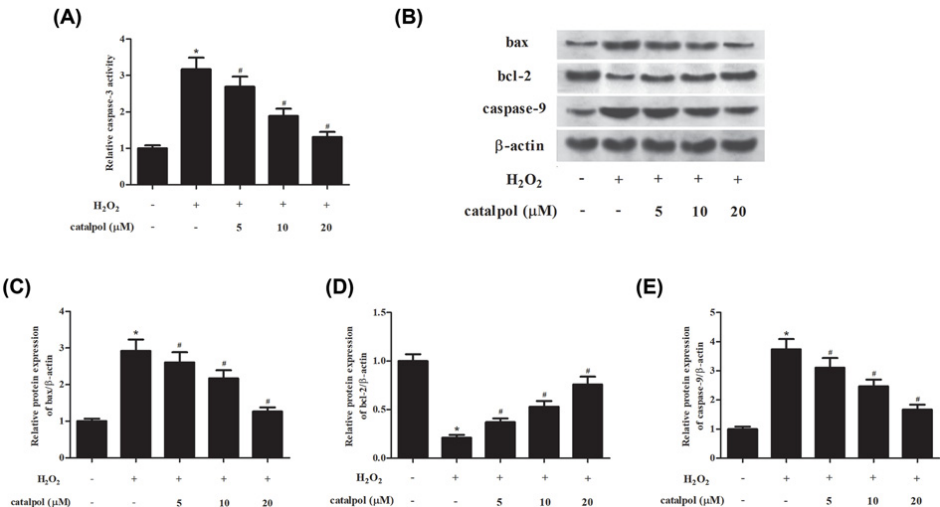
Effect of catalpol on cell apoptosis in H_2_O_2_-stimulated GCs GCs were pretreated with 5, 10 and 20 μM of catalpol for 2 h, and then stimulated with H_2_O_2_ (150 μM) for 24 h. (**A**) The caspase-3 activity was determined using ELISA. (**B**) The expression levels of apoptotic markers (bax, bcl-2 and caspase-9) were examined using Western blot analysis. (**C–E**) Quantification analysis of bax, bcl-2 and caspase-9. *P<0.05 versus the control GCs. ^#^*P*<0.05 versus the H_2_O_2_-stimulated GCs.

Then, we examined the effect of catalpol on caspase-3 activity in H_2_O_2_-stimulated GCs. Results from Figure 4A indicated that caspase-3 activity was dramatically increased in H_2_O_2_-stimulated GCs compared with control cells. However, the induction was restored by treatment with catalpol.

Subsequently, the expression levels of apoptosis-related genes were detected using Western blot analysis. The increased expression levels of bax and caspase-9, as well the decreased expression level of bcl-2 in H_2_O_2_-stimulated GCs were markedly reversed by pretreatment with catalpol (Figure 4B–E).

### Catalpol induced the PI3K/Akt/mTOR pathway in H_2_O_2_-stimulated GCs

**Figure 5:**
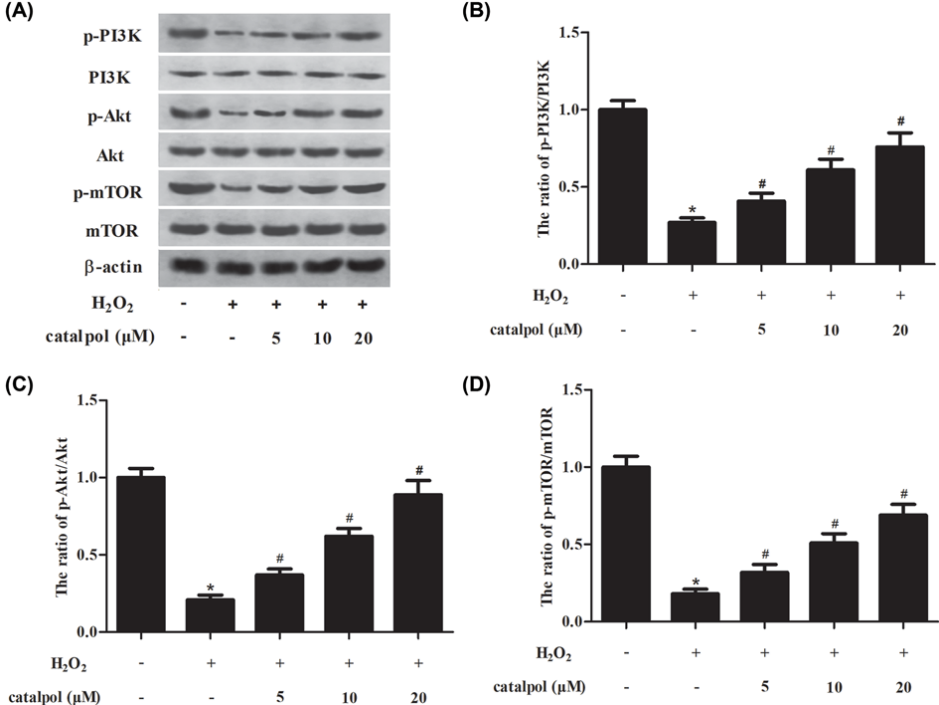
Effect of catalpol on the PI3K/Akt/mTOR pathway in H_2_O_2_-stimulated GCs GCs were pretreated with 5, 10 and 20 μM of catalpol for 2 h, and then stimulated with H_2_O_2_ for 24 h. (**A**) Western blot analysis was performed to detect the expression levels of PI3K, p-PI3K, Akt, p-Akt, mTOR and p-mTOR. (**B**) Quantification analysis of p-PI3K/PI3K. (**C**) Quantification analysis of p-Akt/Akt. (**D**) Quantification analysis of p-mTOR/mTOR. *P<0.05 versus the control GCs. ^#^*P*<0.05 versus the H_2_O_2_-stimulated GCs.

Following detection of the oxidative injury, the mechanism underlying the function of catalpol was explored. Western blot analysis demonstrated that exposure to H_2_O_2_ led to marked decrease in the expression levels of p-PI3K, p-Akt and p-mTOR. However, catalpol-pretreated GCs appeared significant induction in p-PI3K, p-Akt and p-mTOR expressions (Figure 5).

### Inhibition of PI3K/Akt reversed the protective effects of catalpol on GCs

**Figure 6:**
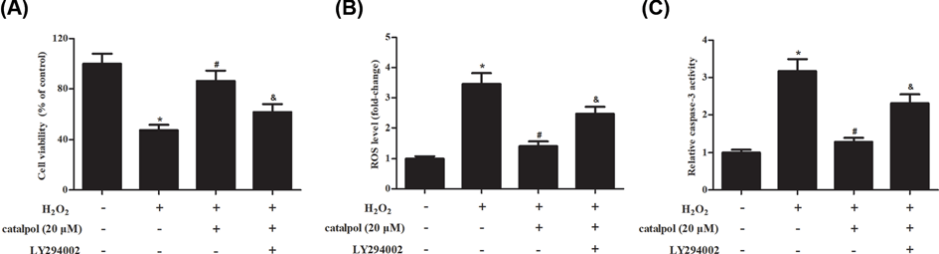
The protective effects of catalpol on H_2_O_2_-stimulated GCs were reversed by LY294002 GCs were treated with LY294002 (10 μM) to inhibit the activation of PI3K/Akt signaling pathway. (**A**) Cell viability was measured using CCK-8. (**B**) ROS production was determined using a fluorescent probe. (**C**) Caspase-3 activity was determined using ELISA. *P<0.05 versus the control GCs. ^#^*P*<0.05 versus the H_2_O_2_-stimulated GCs. ^&^*P*<0.05 versus the catalpol + H_2_O_2_ group.

Next, GCs were treated with LY294002 to inhibit the activation of PI3K/Akt/mTOR signaling pathway. The improved cell viability caused by catalpol (20 μM) was prevented by LY294002 (Figure 6A). Additionally, LY294002 treatment elevated ROS production and caspase-3 activity, as compared with catalpol (20 μM)-pretreated GCs (Figure 6B,C). The results indicated that PI3K/Akt/mTOR signaling pathway mediated the protective effects of catalpol on H_2_O_2_-stimulated GCs.

## Discussion

GCs play crucial roles in folliculogenesis since they can provide nutrients and maturation-enabling factors to sustain oocytes maturation and protect oocytes from oxidative damage [6,15]. GCs apoptosis is a physiological phenomenon in follicle that can trigger follicular atresia, which are responsible for the reduction in follicle numbers [16]. GCs are sensitive to ROS, which are side products formed during citric acid cycle. ROS including superoxide anion radicals, hydroxyl radicals and H_2_O_2_ are markedly increased in response to stimuli and induce oxidative stress in GCs, leading to GCs dysfunction [8]. Therefore, ROS-induced oxidative damage and apoptosis in GCs are considered as the main etiological factor of ovarian insufficiency [8]. In the current study, we used H_2_O_2_ to induce oxidative damage and apoptosis in GCs.

Increasing literatures have demonstrated that catalpol has the capacity to enhance endogenous antioxidant enzymatic activities and inhibit free radical generation. For instance, catalpol markedly reduces LPS-induced production of pro-inflammatory cytokines and ROS in BV2 microglia through inactivating NF-κB signaling [17]. Catalpol suppresses advanced glycation end-products (AGEs)-induced inflammatory responses through inhibition of ROS in human monocytic THP-1 cells [18]. Catalpol protects PreOLs against ischemia-induced oxidative injury via reducing mitochondrial damage and ameliorating overproduction of ROS [11]. Therefore, we aimed to evaluate the protective effect of catalpol on H_2_O_2_-induced oxidative damage in GCs. We found that catalpol significantly improved cell viability of GCs, reduced ROS and MDA production, and elevated SOD and GSH-Px activities, implying that catalpol attenuated oxidative injury in H_2_O_2_-induced GCs.

It has been reported that ROS-mediated oxidative stress may lead to apoptosis via death receptor pathway or mitochondrial pathway [19,20]. In the mitochondrial pathway, bcl-2 family members are activated or inactivated, thereby results of increase in mitochondrial membrane permeability [21,22]. Thereafter, the caspase signaling is activated, which contributes to the execution of apoptosis [23]. Our results showed that catalpol treatment caused increase in bcl-2 and decrease in bax, as well as caspase-9. These findings suggested that catalpol protected GCs from H_2_O_2_-induced oxidative injury and apoptosis.

PI3K/Akt/mTOR is an important signaling pathway that can block cell apoptosis through regulation of downstream signaling molecules, such as inhibiting the activation of caspase-9, as well inactivating bcl-2 family members [24]. It is well known that the PI3K/Akt signaling pathway plays a crucial role in the regulation of GCs growth apoptosis during follicular development [25,26]. Emerging evidence has proven activation of PI3K/Akt signaling pathway protects GCs from oxidative stress and apoptosis [27–29]. Interestingly, previous study has reported that catalpol promotes axonal growth via regulating miR-124 to activate PI3K/Akt/mTOR pathway in neurons after ischemia [30]. Catalpol ameliorates AGEs-induced endothelial dysfunction via suppressing the NF-κB/iNOS pathway and activating the PI3K/Akt/eNOS pathway [31]. However, whether catalpol exerted its functions through PI3K/Akt/mTOR signaling pathway remains to be fully elucidated. In the present study, we demonstrated that catalpol caused significant activation of PI3K/Akt/mTOR pathway in GCs in response to H_2_O_2_ stimulation. Furthermore, inhibition of PI3K/Akt/mTOR blocked the protective effects of catalpol on H_2_O_2_-induced oxidative injury and apoptosis in GCs, which suggested that the protective effects of catalpol were mediated by PI3K/Akt/mTOR signaling pathway.

There existed several limitations in the present study. First, we only evaluated the effect of catalpol on rat GCs *in vitro*. An *in vivo* animal study will be considered in the following studies. Second, whether catalpol can be translated in clinical application will require further experiments.

Based on the findings of the present study, we concluded that catalpol has protective effects on H_2_O_2_-induced oxidative injury and apoptosis in GC. These protective effects may be mediated by activation of PI3K/Akt/mTOR signaling pathway. The results indicated that catalpol might be a therapeutic agent for ovarian pathologies through regulation follicular development.

## Abbreviations

AGE: advanced glycation end-product
CCK-8: cell counting kit-8
DCF: 2′, 7′–dichlorofluorescein
eNOS: endothelial nitric oxide synthase
GC: granulosa cell
GSH-Px: glutathione peroxidase
H2-DCFDA: 2′,7′-Dichlorodihydrofluorescein diacetate
H_2_O_2_: hydrogen peroxide
iNOS: inducible nitric oxide synthase
LDH: lactate dehydrogenase
MDA: malondialdehyde
PreOL: pre-myelinating oligodendrocyte
ROS: reactive oxygen species
SOD: superoxide dismutase
